# Cell Transformation by RNA Viruses: An Overview

**DOI:** 10.3390/v3060858

**Published:** 2011-06-15

**Authors:** Hung Fan

**Affiliations:** Department of Molecular Biology and Biochemistry, Cancer Research Institute, University of California Irvine, Irvine, CA 92697, USA; E-Mail: hyfan@uci.edu; Tel.: 1-949-824-5554; Fax: +1-949-4023

Studies of oncogenic viruses have made seminal contributions to the molecular biology of cancer. Key discoveries include the identification of viral oncogenes and cellular proto-oncogenes, elucidation of signal transduction pathways, and identification of tumor suppressor genes. The origins of cancer virology began almost exactly one hundred years ago with the discovery of avian sarcoma and acute leukemia viruses—RNA-containing viruses of the retrovirus family. The study of animal cancer viruses accelerated beginning in the late 1960s and early 1970s, with the discovery of DNA viruses that could transform cells in culture, and the development of quantitative assays for transformation by DNA and RNA-containing tumor viruses. The discovery of reverse transcriptase in retroviruses in 1970 also greatly accelerated research on these viruses. Indeed RNA and DNA tumor viruses led the way in cancer molecular biology during this era before molecular cloning. It was possible to physically purify virus particles and generate specific hybridization probes for viral DNA and RNA at a time when it was not possible to analyze cellular genes in the same manner.

Laboratory studies on RNA and DNA tumor viruses (many of them animal viruses) are highly relevant to human cancer. First, many of the principles of viral oncogenesis elucidated by these studies are directly applicable to human cancers, including those that are not caused by viruses. In addition, a significant percentage of human cancers worldwide (∼15%) have a viral involvement. Human DNA tumor viruses include human papillomavirus (HPV, high risk strains), Merkel cell polyomavirus (MPV), the gammaherpesviruses Epstein-Barr virus (EBV) and Kaposi’s sarcoma herpesvirus (KSHV), and hepatitis B virus (HBV). Human RNA viruses that cause cancer include the retrovirus Human T-cell Leukemia virus type I (HTLV-I) and the flavivirus hepatitis C virus (HCV). In addition, AIDS is caused by a retrovirus (HIV-1 and -2); a major complication of the immunodeficiency characteristic of AIDS is the development of cancers. Most of these cancers have an underlying viral involvement (e.g. KSHV and Kaposi’s sarcoma, which was first discovered in AIDS patients).

The articles in this issue are part of a planned larger collection that will also include chapters on DNA tumor viruses, to be published at a later date. The bulk of the contributions focus on retroviruses, since they have been actively studied for more than fifty years. Oncogenic retroviruses can be divided into acute transforming viruses that carry viral oncogenes and induce tumors rapidly, *vs.* non-acute retroviruses that do not carry oncogenes and which induce tumors more slowly. The prototypic acute transforming retrovirus is Rous sarcoma virus (RSV) which carries the *v-src* oncogene. *V-src* was derived from the cellular proto-oncogene *c-src*. The other acute transforming retroviruses also carry captured versions of cellular proto-oncogenes. As a class the cellular proto-oncogenes stimulate cell growth or division, and the viral oncogenes derived from them constitutively stimulate cell growth or division by employing the same pathways. Acute transforming retroviruses and their oncogenes were intensively studied in the past, after which attention shifted to the normal functions and regulation of the cellular proto-oncogenes. Since they have been extensively reviewed in the past [1], they were not included in this issue.

Non-acute retroviruses typically induce tumors by transcriptionally activating cellular protooncogenes; this generally results from influence of the viral long terminal repeats (LTRs) on the protooncogenes—LTR activation of proto-oncogenes. These mechanisms, and other mechanisms of oncogenesis by non-acute retroviruses are reviewed in the chapter by Fan and Johnson [2]. Implications of non-acute retroviral oncogenesis to safety in gene therapy trials are also discussed.

The other chapters on retroviruses summarize work on some of the oncogenic retroviruses that are under current investigation. Cmarik and Ruscetti [3] review leukemogensis by the Friend MuLV complex, which consists of an acute transforming virus SFFV as well as a helper virus (F-MuLV). Friend SFFV is interesting because its oncogene is a deleted version of a retroviral envelope protein that is a recombinant between F-MuLV and an endogenous MuLV-related provirus. The mechanisms by which the SFFV env protein activates signal transduction pathways have been well-studied and are reviewed. The Friend virus complex has also provided substantial insights on insertional oncogenesis, including inactivation of the p53 tumor suppressor gene. Ross [4] reviews replication and oncogenesis by murine mammary tumor virus (MMTV). MMTV initially infects cells of the immune system (dendritic cells, T and B lymphocytes) before trafficking to the mammary gland where insertional activation of proto-oncogenes and mammary carcinogenesis occurs. MMTV is also interesting because it encodes additional proteins (besides the standard retroviral Gag, Pol and Env proteins), including a viral superantigen (Sag) and a small regulatory protein Rem that are both important for replication *in vivo*. Studies of MMTV have also provided insight into a host restriction factor APOBEC3. Jaagsiekte sheep retrovirus replication and oncogenesis is reviewed by Hofacre and Fan [5]. JSRV (like MMTV) is a betaretrovirus, and it induces a transmissible lung cancer in sheep. While JSRV has not captured a cellular proto-oncogene, the envelope protein of this virus also functions as an oncogene. The mechanisms by which JSRV Env induces oncogenic transformation are discussed. Like MMTV, JSRV also encodes a small regulatory protein Rej that is necessary for efficient translation of unspliced viral RNA.

Rovnak and Quackenbush [6] review oncogenesis by epsilonretroviruses, in particular walleye dermal sarcoma virus (WDSV). WDSV causes dermal sarcomas in walleye pike; there is striking seasonal variation in that the tumors occur during the winter, and they slough off during the springtime. The tumors express very little infectious virus, but virus production occurs during the spring as tumors are being sloughed. WDSV encodes additional proteins, one of which is a viral cyclin. This cyclin appears to function as both a repressor of viral transcription and an oncogene.

Kannian and Green [7] review replication and oncogenesis by HTLV-I. HTLV-I and other deltaretroviruses encode a series of additional proteins derived by alternate splicing into the *X* region of the genome. The mechanisms of function of these proteins and their potential roles in oncogenesis are described. It is noteworthy that leukemogenesis by HTLV-I is quite inefficient, with a low percentage of infected people ultimately developing disease, after decades. The roles of the viral Tax and HBZ proteins in oncogenesis are summarized.

The other RNA virus associated with cancer is hepatitis C virus (HCV), reviewed by Banerjee *et al.* [8]. A noteworthy feature of HCV infection *in vivo* is that a significant fraction of individuals infected by HCV are unable to clear the infection. The persistent infection can lead to chronic liver damage and ultimately development of hepatocellular carcinoma. It is interesting that the unrelated HBV can also establish persistent infection and those individuals are also at risk for developing liver cancer. In this article the potential roles of several viral proteins in causing cellular damage that could lead to tumorigenesis in persistently infected individuals are described.
